# Global Trade, Local Impacts: Lessons from California on Health Impacts and Environmental Justice Concerns for Residents Living near Freight Rail Yards

**DOI:** 10.3390/ijerph110201914

**Published:** 2014-02-10

**Authors:** Andrea Hricko, Glovioell Rowland, Sandrah Eckel, Angelo Logan, Maryam Taher, John Wilson

**Affiliations:** 1 Southern California Environmental Health Sciences Center, Department of Preventive Medicine, Keck School of Medicine, University of Southern California, 2001 N. Soto Street, Los Angeles, CA 90089, USA; E-Mails: growland@usc.edu (G.R.); eckel@usc.edu (S.E.); 2 East Yard Communities for Environmental Justice, 2317 Atlantic Boulevard, Commerce, CA 90040, USA; E-Mail: alogan@eycej.org; 3 Spatial Sciences Institute, Dornsife College of Letters, Arts and Sciences, University of Southern California, AHF B55, Los Angeles, CA 90089, USA; E-Mails: mtaher@usc.edu (M.T.); jpwilson@usc.edu (J.W.)

**Keywords:** air pollution, diesel exhaust, environmental health, environmental justice, exposure, health disparities, international trade, land use, particulate matter, race/ethnicity, rail

## Abstract

Global trade has increased nearly 100-fold since 1950, according to the World Trade Organization. Today, major changes in trade are occurring with the advent of mega-ships that can transport thousands more containers than cargo ships now in use. Because global trade is expected to increase dramatically, the railroad industry—in the U.S. alone—has invested more than $5 billion a year over the past decade to expand rail yards and enhance rail routes to transport goods from ports to retail destinations. This article describes cancer risks for residents living in close proximity to rail yards with emissions of diesel particulate matter pollution from locomotives, trucks and yard equipment. The article examines the demographics (income, race/ethnicity) of populations living in the highest estimated cancer risk zones near 18 major rail yards in California, concluding that the majority are over-represented by either lower-income or minority residents (or both). The authors also describe a review of the news media and environmental impact reports to determine if rail yards are still being constructed or expanded in close proximity to homes and schools or in working class/working poor communities of color. The paper suggests policy efforts that might provide more public health protection and result in more “environmentally just” siting of rail yards. The authors conclude that diesel pollution from rail yards, which creates significant diesel cancer risks for those living near the facilities, is an often overlooked public health, health disparities and environmental justice issue in the U.S. The conclusions are relevant to other countries where international trade is increasing and large new intermodal rail facilities are being considered.

## 1. Introduction

In the U.S., major freight railroads are making record investments in infrastructure, with more than a dozen new rail yard facilities built or proposed during the past few years across the country in anticipation of increased international trade [1]. Part of the railroads’ impetus in these investments is the Panama Canal expansion, expected to be completed in 2015, which will allow the world’s largest container ships, for the first time, to pass through the Canal. Many ports are expanding their operations or even dredging their harbors so that they can be competitive in attracting the larger ships, especially from Asia, once the new locks on the Canal are finished [2]. In response, the largest freight railroad companies are building or expanding major rail facilities both near ports and further inland to handle the transfer of containers filled with goods, made mostly in China and other Asian countries, between one mode of transportation (e.g., trucks) to another (e.g., trains)—a process referred to as “intermodal” rail [3]. Ports and rail operations are expanding in other countries, as well, in anticipation of mega-container ships and increased trade potential [4].

Intermodal rail facilities employ significant amounts of diesel-fueled equipment, including line-haul (cross-country) locomotives, switch engines (which stay in the rail yards), cranes, and yard equipment. After arriving from Asia on ships, containers most often are trucked by heavy-duty diesel trucks emitting diesel particulate matter or moved by trains with diesel-fueled locomotives to their destinations. Concerns about the health effects of exposure to diesel exhaust emissions have been raised for decades [5]. In 2002, the U.S. Environmental Protection Agency (EPA) diesel assessment stated that “long-term (*i.e.*, chronic) inhalation exposure is likely to pose a lung cancer hazard to humans, as well as damage the lung in other ways depending on exposure [6]”. In 2012, the International Agency for Research on Cancer, part of the World Health Organization, went further by classifying diesel engine exhaust as “carcinogenic to humans (Group 1), based on sufficient evidence that exposure is associated with an increased risk for lung cancer [7]”. A recent study estimated that 6% of all lung cancer deaths in the U.S. and United Kingdom are related to diesel exhaust exposure, including in the workplace and general population [8].

Some rail yards in the U.S. were built decades (or even a century) ago; others were built more recently or within the past 30 years. Today, many residents live in close proximity to many of these older yards, raising concerns about exposure to traffic-related air pollution, including from diesel-fueled trucks hauling containers in and out of rail yards. A recent study in the southeastern United States found that rail yard emissions led to increases of particulate matter and black carbon (as a marker for diesel emissions) [9]. A study of a rail yard in northern California found emissions of particulates, sulfur dioxide, metals and polycyclic aromatic hydrocarbons [10]. Meanwhile, the body of research evidence is growing that shows adverse health effects from living or going to school in close proximity to traffic-related air pollution. These include effects such as reduced lung function in exposed children [11], increased asthma prevalence and incidence [12,13,14]; effects in pregnant women [15] and their offspring (e.g., premature births [16]); harmful effects in adults and the elderly including possibly cognitive decline [17] and heart attacks [18]; and more. 

Because of such studies and concerns raised by residents and community groups, the California Air Resources Board (CARB) has developed guidelines for siting new residences, schools, day care centers, playgrounds, and medical facilities (*i.e.*, sensitive receptors) near certain types of operations, including highways and rail yards, among others. The guidelines state the following about rail yards:

“*We recommend doing everything possible to avoid locating sensitive receptors within the highest risk zones at ports and rail yards… Avoid siting new sensitive land uses within 1,000 feet of a major service and maintenance rail yard. Within one mile of a rail yard, consider possible siting limitations and mitigation approaches*”.

[19]

The CARB guidelines operate in only one “direction” when land use decisions are made. They suggest how far away schools and other “sensitive receptors” (e.g., facilities for children, the elderly and the ill) should be located from ports/rail yards/highways. But they do not make recommendations for land use decisions that would site new highway, port or rail facilities near these same types of sensitive land uses, such as schools. The guidelines specify that one should avoid siting a school near a rail yard, but are silent on whether it is acceptable to site a rail yard in close proximity to a school, with CARB deferring to local government authorities on that issue. Thus, railroads are able to claim that the CARB guidelines do not pertain to them when siting new intermodal facilities in California.

Community-based groups and residents in California have been calling for stricter regulations on locomotive and rail yard pollution for nearly 10 years [20]. Although California has been more active than other states in trying to reduce diesel exhaust from rail yards, in that state only *voluntary* agreements have been negotiated between the freight railroads and CARB to reduce diesel particulate matter pollution [21], with CARB arguing that Federal laws protect the railroads from state regulations. Language in a 2005 agreement required CARB to produce a HRA for each of the 18 major rail yards in the state, based on emissions inventories provided by the major freight railroads. In CA, these railroads include only BNSF and UP, which are the two largest freight railroads in the country. CARB completed the last of the rail yard HRAs in 2008 [22].

The state’s largest rail yards are located in southern California, and these have been the focus of significant attention by residents, environmental and community organizations and a community-academic collaborative called THE Impact Project [23,24], all calling for a reduction in diesel emissions to protect public health [25]. Some of the community-based groups in southern California have held educational rallies to inform others about the diesel cancer risks [26] and have called for stricter regulation of diesel locomotives and for rules on rail yard emissions [27]. Members of these groups express concern about disproportionate impacts and “environmental justice” (EJ), which the U.S. Environmental Protection Agency defines as:

“*... the fair treatment and meaningful involvement of all people regardless of race, color, national origin, or income with respect to the development, implementation, and enforcement of environmental laws, regulations, and policies.*”

[28]

Many investigators have conducted EJ research, examining whether specific groups are more highly exposed to pollution when compared to other racial/ethnic/income groups [29,30,31]. Some studies in California have looked at environmental justice and air toxics [32]; EJ and drinking water contamination [33]; and the disproportionate presence of liquor stores in certain neighborhoods [34].

This is the first published study, to our knowledge, to assess issues of race and income near California’s 18 major rail yards and to determine if residents are correct about their perceived claims of disproportionate impacts for lower-income and minority residents living near the facilities. The authors review the HRAs for the state’s 18 major rail yards and analyze the demographics of residents living near them, which were not examined by CARB staff, in order to assess potential racial and economic disparities. Ten years ago, an analysis was commissioned by the U.S. Environmental Protection Agency (EPA) to support its locomotive engine rule. That analysis investigated the populations living in close proximity to a representative sample of 37 U.S. rail yards, including three yards in California [35].

We also review news media and trade journal articles to determine whether environmental justice and disproportionate impacts, as well as proximity to homes and schools, are considerations in the siting of new rail yard facilities around the country, and we offer some alternatives for what might constitute “environmentally just” siting.

Overall, our objectives in this paper are to:

- Describe the number of California residents who live in the zones of highest diesel cancer risk near existing rail yards in the state and determine if there are racial/ethnic and income disparities among them;
- Determine through a review of the news media and trade journals whether new or expanding rail yards are taking into consideration the proximity of schools and homes to the newly proposed sites, as well as the potential for disproportionate impacts; and
- Offer insights into what makes an intermodal rail yard unique in terms of industrial facilities and what types of considerations are needed to help ensure that rail yard siting or expansion takes community, public health and environmental justice concerns into account.

## 2. Background Information from the California Air Resources Board Health Risk Assessments

Between 2005–2008, the California Air Resources Board (CARB) published a Health Risk Assessment (HRA) for each of the 18 major rail yards in California (CA) [36], using guidance from the California Office of Health Hazard Evaluation and Assessment (OEHHA) [37,38]. These HRAs looked at diesel particulate emissions from locomotives, cranes and yard equipment within the rail yard boundaries and also onsite and offsite emissions from heavy duty diesel-powered trucks that take containers to and from the rail yards.

### 2.1. Diesel Emissions at 18 California Rail Yards

The CARB HRAs evaluated (through modeling efforts) the potential health risks associated with diesel particulate matter (DPM) emissions to those living nearby the rail yards, considering “the rail yard property emissions from locomotives, on-road heavy-duty trucks, cargo handling equipment, and off-road equipment used to move bulk cargo; also evaluated were mobile and stationary sources with significant emissions within a one-mile distance of the rail yard”. The estimates were based on 2005 emissions. Emissions from each individual yard ranged from a low of 1.7 annual tons to a high of 27.9 annual tons (Table 1, adapted from CARB HRA) [39]. CARB noted that residents of Commerce, CA, which has a population of 13,000, face particularly serious impacts because there are four rail yards located in that single community, with combined DPM emissions totaling more than 40 tons per year. Because of this, CARB decided to do a separate HRA for these four combined yards [40].

Both the railroad companies and the California Air Resources Board state that there has been a significant reduction in diesel particulate emissions at these four yards since the voluntary agreement and the HRAs were released in 2007–2008 [41]. In January 2014 CARB announced that it had decided to start using a different approach to try to obtain emission reductions at rail yards by no longer pursuing voluntary agreements with the railroad companies but instead developing a “Sustainable Freight Transport Initiative that will outline the needs and steps to transform California’s freight transport system to one that is more efficient and sustainable,” one that will “move goods more efficiently and with zero/near-zero emissions… and support healthy, livable communities” [42].

### 2.2. CARB’s Development of Isopleths (Contour Lines or Zones) for Diesel Cancer Risk around the Rail Yards

**Figure 1:**
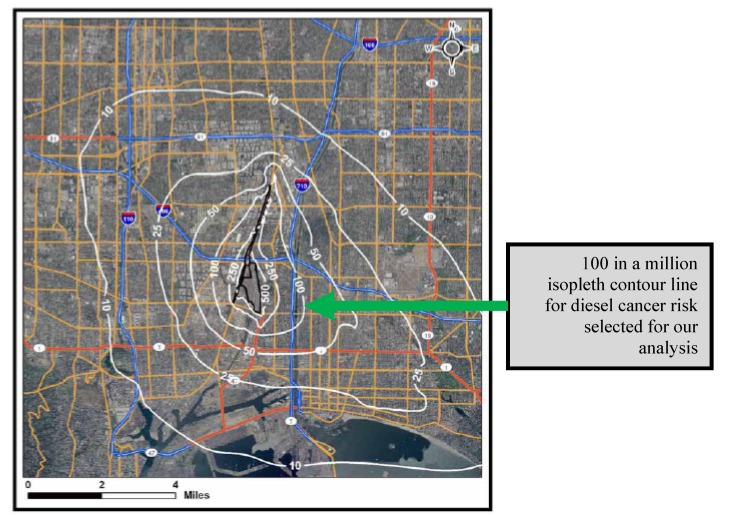
Estimated potential cancer risks (chances per million) associated with diesel particulate matter emissions at the Union Pacific Intermodal Container Transfer Facility (UP ICTF) in Wilmington, CA *.

CARB developed isopleths for diesel cancer risk around the 18 rail yards. The agency defined an isopleth as a “*line drawn on a map through all points of equal value of some measurable quantity; in this case, cancer risk*”. That complicated statement translates, in this case, as a “contour line” or “zone” that delineates the estimated average potential cancer risk near the rail yard property boundaries, assuming a 70-year exposure [37,38]. Using one rail yard, the Union Pacific Intermodal Container Transfer Facility (UP ICTF), as an example, we show below the isopleths developed by CARB for the estimated average potential cancer risk of 100 chances per million in close proximity to the rail yard property boundaries [43], Figure 1. The risks decrease the further away from the rail yard one lives (with wind patterns taken into consideration). For example, as seen in Figure 1, residents who live three miles away from the rail yard are primarily within the 10 in a million to 25 in a million cancer risk zones or isopleths.

* Reproduced with permission of the California Air Resources Board.

**Table 1:** Tons of annual diesel particulate matter emissions from the 18 rail yards in California by source of emissions, from Health Risk Assessments published by CARB during 2005–2008, listed in descending order by total emissions estimated in 2005 ^a^.

Rail Yard	Locomotives	Cargo Handling Equipment	On- Road Trucks	Other (Off-Road Equipment, Transport Refrigeration Units, Stationary Sources, *etc*.)	Total (Tons)
**Commerce: 4 yards** **combined **	13.6	9.4	13.2	5.5	41.8
**BNSF Barstow** **^a^**	27.1	0.03	0.04	0.75	27.9
**UP Roseville**	25.1	N/A	N/A	N/A	25.1
**BNSF Hobart** **^b^**	5.9	4.2	10.1	3.7	23.9
**UP ICTF/Dolores**	9.8	4.4	7.5	2.0	23.7
**BNSF San Bernardino**	10.6	3.7	4.4	3.4	22.0
**UP Colton**	16.3	N/A	0.2	0.05	16.5
**UP Commerce** **^b^**	4.9	4.8	2.0	0.4	12.1
**UP Oakland**	3.9	2.0	1.9	3.4	11.2
**UP City of Industry**	5.9	2.8	2.0	0.3	10.9
**UP LATC**	3.2	2.7	1.0	0.5	7.3
**UP Stockton**	6.5	N/A	0.2	0.2	6.9
**UP Mira Loma**	4.4	N/A	0.2	0.2	4.9
**BNSF Richmond**	3.3	0.3	0.5	0.6	4.7
**BNSF Stockton**	3.6	N/A	N/A	0.02	3.6
**BNSF Commerce Eastern** **^b^**	0.6	0.4	1.1	1.0	3.1
**BNSF Sheila** **^b^**	2.2	N/A	N/A	0.4	2.7
**BNSF Watson**	1.9	N/A	<0.01	0.04	1.9
**BNSF San Diego**	1.6	N/A	0.007	0.04	1.7

^a^ Please note that this does not necessarily mean that the residents near these yards have the highest cancer risk of the 18 yards, because other yards may have residents living in closer proximity or have wind patterns that blow emissions into their communities. For example, the BNSF Barstow rail yard has the highest annual emissions, but the BNSF San Bernardino yard has the highest diesel cancer risk for nearby residents. ^b^ Railyards with this notation are located in the City of Commerce.

### 2.3. Exposed Populations (and Their Estimated Cancer Risks) near the Four Highest Priority Rail Yards in California

Based on its Health Risk Assessment analyses, the California Air Resources Board calculated the number of persons exposed at different diesel cancer risk levels at each of the 18 rail yards. From the 18 yards, CARB identified four with particularly high estimated diesel cancer health risks, as seen in Table 2 [44]. Table 2 shows the number of residents estimated to be exposed to certain levels of risk within the described zones of diesel cancer risk (isopleths) at these four yards. In addition, based on the HRAs, CARB identified residents of the City of Commerce as heavily impacted by diesel emissions and cancer risk because Commerce has four rail yards within its boundaries [40], so the combined four yards are also shown in the Table. All of these rail yards are in southern California and serve the Ports of Los Angeles (L.A.) and Long Beach, the largest ports in the U.S. Note the large number of individuals exposed to greater than 500 in one million risk in both San Bernardino [39] and Wilmington [43]. Of all rail yards, the BNSF San Bernardino had the highest population exposure to rail yard emissions, due to significant emissions and the large number of residents living nearby. At this yard, CARB found that 3,780 residents had an estimated cancer risk averaging 980 chances per million, meaning that if residents near the yard were exposed to diesel emissions at that level for a 70-year lifetime, 500 in a million would be expected to develop cancer [39]. Table 2 also shows the estimated diesel cancer risk for residents near the four combined yards in the City of Commerce, where an estimated 5,200 residents have a potential cancer risk averaging 690 in a million [40].

### 2.4. Proximity of Homes and Schools to the Top Four Highest Priority Rail Yards in California

For its HRAs, the California Air Resources Board used GoogleMaps to determine whether homes, parks and/or schools were in close proximity to the 18 rail yards. The text below describes the “sensitive receptors” (homes, schools, hospitals) that CARB described as being near the four rail yards in California with the highest levels of diesel cancer risk and population exposed.

*UP Commerce Rail Yard*: Within two miles of this yard, there are 27 sensitive receptors, including 19 schools, four child care centers and four hospitals. Four of these sensitive receptors are within the 100 in a million cancer risk range. Homes are adjacent to the rail yard fence, and an elementary school is located less than two blocks away [45].
*BNSF Hobart Yard, Commerce*: CARB looked at sensitive receptors within a two-mile distance of the yard and found 28, including eight schools, 12 child care centers and eight hospitals. Within the 100 in a million cancer risk range, there were 19 sensitive receptors identified [46].
*UP ICTF, Wilmington*: The UP ICTF is just 400 feet away from a middle school and homes that are located in west Long Beach, CA. There are seven sensitive receptors in the 100 in a million cancer risk range and 20 sensitive receptors all located within one mile of the rail yard [43].
*BNSF San Bernardino*: Homes are located directly across the street from this yard. Within a one-mile distance of the yard, there are 41 sensitive receptors, including seven hospitals/medical centers, 19 childcare centers and 15 schools. When considering a 100 in a million cancer risk range, there are 19 sensitive receptors [39].

**Table 2:** Estimated exposed populations associated with different cancer risk levels (assuming a 70 year exposure) near the most impacted rail yards in California, listed in order by the highest number of residents exposed to a cancer risk of greater than 500 in one million *.

Rail Yard	Estimated Population Exposed to Cancer Risk of Greater than 100 Chances in a Million	Estimated Population Exposed to Cancer Risk of Greater than 500 Chances in a Million
**4 yards in Commerce combined**	82,000	5,200
**BNSF, San Bernardino**	39,580	3,780
**UP ICTF, Wilmington**	33,540	1,200
**BNSF Hobart, Commerce**	48,200	100
**UP, Commerce**	12,000	100

* Data compiled from individual California Air Resources Board’s Health Risk Assessments.

## 3. Study Methods

As published, the HRAs contained no analysis of demographic information about residents living in the vicinity of the rail yards. We employed Geographic Information Systems (GIS) to study the demographics of residents facing high calculated diesel cancer risks in close proximity to the 18 rail yards, and we compared them to demographics of the entire county in which the residents live. To accomplish this, we examined each HRA’s estimates of population exposure, as well as cancer risk isopleths (contours) showing areas where residents are at greater risk of exposure to diesel particulate emissions (DPM) and diesel cancer risk as calculated by CARB. For cancer impacts, CARB plotted total risk isopleths for facilities in the HRAs at potential cancer risk intervals of 1, 10, 25, 50, 100, 250, 500, *etc*. in a million. We selected the 100 in a million risk as our definition of impacted nearby residents, because most (but not all) rail yards had residents living within that risk isopleth. At higher risk levels (250 or 500 in a million), some rail yards had few residents within the isopleths. In doing this, we were able to have consistent risk levels to compare across most of the 18 rail yards. Figure 1 shows an example of an isopleth (contour) from an HRA. Using the isopleths and maps in the HRAs, which we digitized, we focused on the race/ethnicity and annual incomes of residents within isopleths that had high cancer risks (which we defined as “100 or more chances in a million”) and compared them to the same variables within the county of residence. We retrieved and analyzed data from the 2000 census at the census block group level to look at race, ethnicity and income levels, in an effort to determine if there were diesel cancer risk disparities and environmental justice concerns at any of the 18 major rail yards in California. Where needed (*i.e.*, when isopleths crossed two or more counties), we apportioned the results between two counties. Finally, we extracted and used the estimates provided by Ethington and colleagues [47] for these same characteristics for the Intermodal Container Transfer Facility and Dolores Railyard in 1980 for our case study, which involves a rail facility proposed in 1982 [48], built in 1986, and proposed for expansion in 2005. Ethington *et al*. used the 1970–1980–1990 correspondence tables published by the California Department of Finance in 1996 [47] to reassign the census variables from the first two censuses to 1990 Census units, and we then used spatial analysis tools inside ArcGIS^TM^ (ESRI, Redlands, California, USA) to assign these totals to 2000 Census units.

Using a two-sided Pearson’s chi-square test, we tested whether the proportion of non-white residents in a given risk isopleth was equal to the proportion of non-white residents in the county in which the rail yard was located (“population proportion”). White was defined as non-Hispanic white. To further understand racial/ethnic differences, we calculated the proportion of African-American (non-Hispanic Black or African American) and the proportion of Hispanic residents in an isopleth and graphically compared these values (along with their 95% confidence intervals) to the corresponding population proportions. Next, we used a two-sided Pearson’s chi-square test to test whether the proportion of low income households (<$30,000/year) in each isopleth was equal to the population proportions. We plotted these estimated proportions by rail yard, along with their 95% confidence intervals. For 16 of the 18 rail yards, we used 100 in a million risk isopleths. No demographic data was available in the 100 in a million risk isopleth for UP Roseville and UP Mira Loma, likely due to the small number of residents in these small isopleths. Instead, we used a 50 in a million risk isopleths for these rail yards.

Many of these 18 rail yards were sited decades ago, so it is difficult to determine whether the existing rail yards were built first or if the community might have settled there before the yard was built. One of the rail yards, the Union Pacific ICTF, was proposed in 1982 [48] and opened in 1986, so we were able to examine the demographics around that yard using Census data for 1980. In 2005, Union Pacific announced that it wanted to expand its existing ICTF [49], so we also examined more recent demographics using 2000 Census data.

Finally, we conducted a review of the news media and key industry trade journals from 2009 to the present to identify new intermodal rail facilities proposed to be built or recently constructed in the U.S. We reviewed the articles to determine if any of the rail facilities that were recently built or proposed to be built are sited in close proximity to homes and schools or adjacent to neighborhoods that are lower-income and minority.

## 4. Study Site

**Figure 2:**
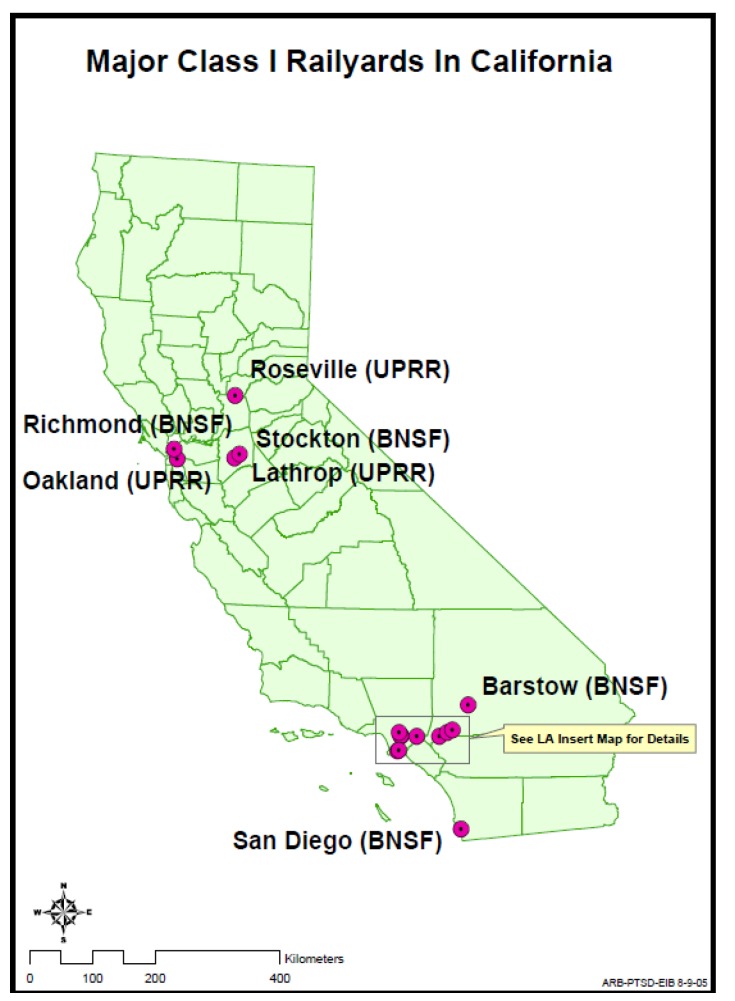
Map* showing locations of the 18 rail yards in California for which CARB conducted HRAs.

**Figure 3:**
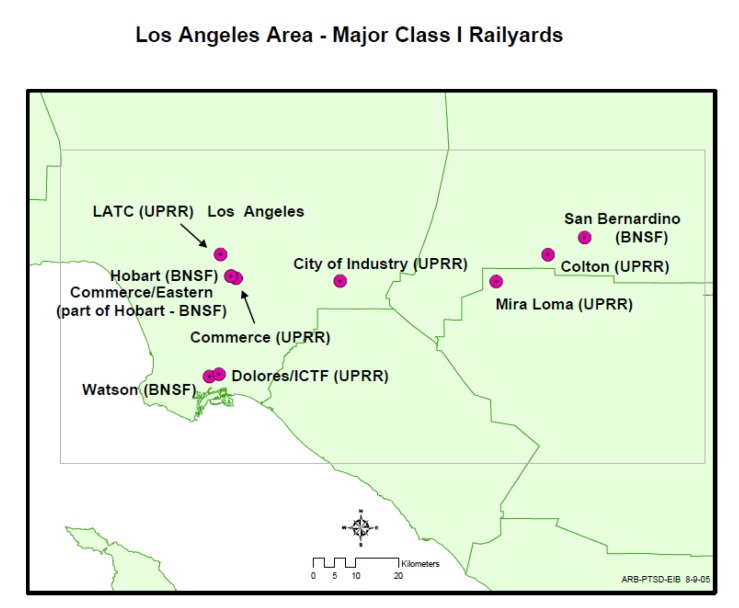
Map* showing locations of the rail yards in the Los Angeles area of California for which CARB conducted HRAs.

Our primary study site was California, with a focus on southern California. Figure 2 shows the location of the 18 major rail yards in California, for which CARB conducted HRAs. Figure 3 shows an inset map for rail yards in the Los Angeles area [36].

* Reproduced with permission of the California Air Resources Board.

* Reproduced with permission of the California Air Resources Board.

## 5. Results

At just the three highest priority rail yards in CA coupled with the four combined rail yards in Commerce, more than 167,000 residents had an estimated diesel cancer risk of greater than 100 in a million, Table 2. With regard to race/ethnicity, 17 of the 18 yards had a statistically significantly *higher* percentage of non-white residents in the high risk cancer isopleths near the rail yard than the population percentage in the respective county (p < 0.0001). Of these, 16 had demographic data for the 100 in a million isopleth risk zone and one (UP Mira Loma) only for the 50 in a million ispopleth risk zone. For UP Roseville, using a 50 in a million risk isopleth, the percentage of non-white residents was statistically significantly *lower* than the population percentage in the County (23% *vs.* 38%, *p* < 0.0001).

**Figure 4:**
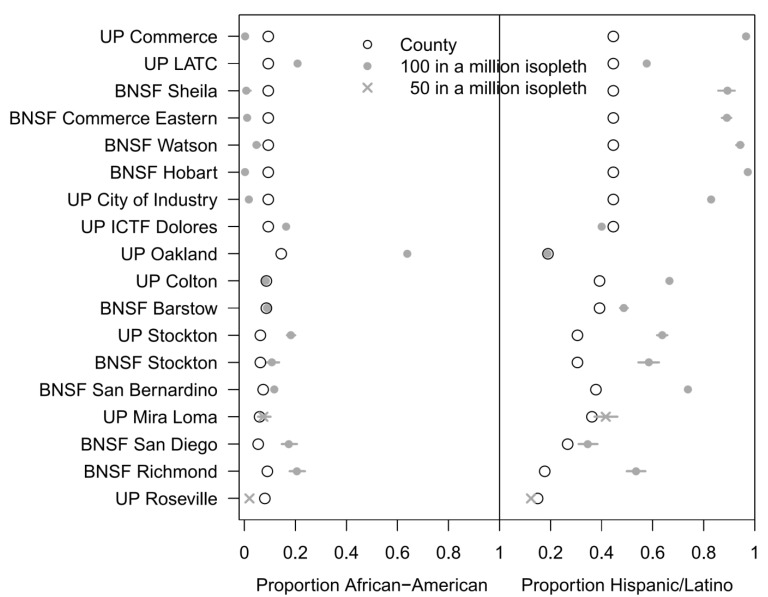
Estimated proportion ^1^ of African American or Hispanic/Latino ^2^ residents living in close proximity to rail yards, where proximity is defined by a 50 in a million risk isopleth or 100 in a million risk isopleth, compared to the corresponding population proportions in the county in which the rail yard is located, Table S1. The rail yards are listed in decreasing order of population percentage non-white in the County,^ 3^ Table S2.

^1^ Point estimates are plotted along with 95% confidence intervals, though for most isopleths the sample size is large enough that the confidence intervals are very narrow and not visible at this scale. ^2^ We use Latino and Hispanic interchangeably in this paper. ^3^ UP ICTF is the only rail yard in the state that also has a high percentage of Asian/Asian Pacific Islanders in the nearby population, compared to the percentages in the county, which is not reflected in this figure.

Our analysis found that the percentage of Latino residents in close proximity to a rail yard was generally much higher than the corresponding population percentage in the respective county, while the pattern was less consistent for African-American residents, Figure 4. For several rail yards (e.g., BNSF Hobart) the percentage Latino in the 100 in a million risk isopleth was extremely high (BNSF Hobart: 97%), resulting in a percentage African-American in the risk isopleth that was lower than the population percentage African American in the county (BNSF Hobart: percentage in the 100 in a million risk isopleth was 0.3% while population percentage in the county was 9.4%). For UP Oakland, the percentage Latino in the 100 in a million risk isopleth was similar to the population percentage (100 in a million risk isopleth: 19%, county: 19%), but the percentage African-American in close proximity was strikingly higher than the population percentage in the respective county (100 in a million risk isopleth: 64%, county: 14%). For UP Roseville, the percentages Latino and African-American were lower in close proximity to the rail yard than the corresponding population percentages (50 in a million risk isopleth: 12%, county: 14%; and 50 in a million risk isopleth: 2%, county: 8%, respectively). See Table S1 for County population percentages non-white and Table S2 for demographic details and 95% confidence intervals. 

**Figure 5:**
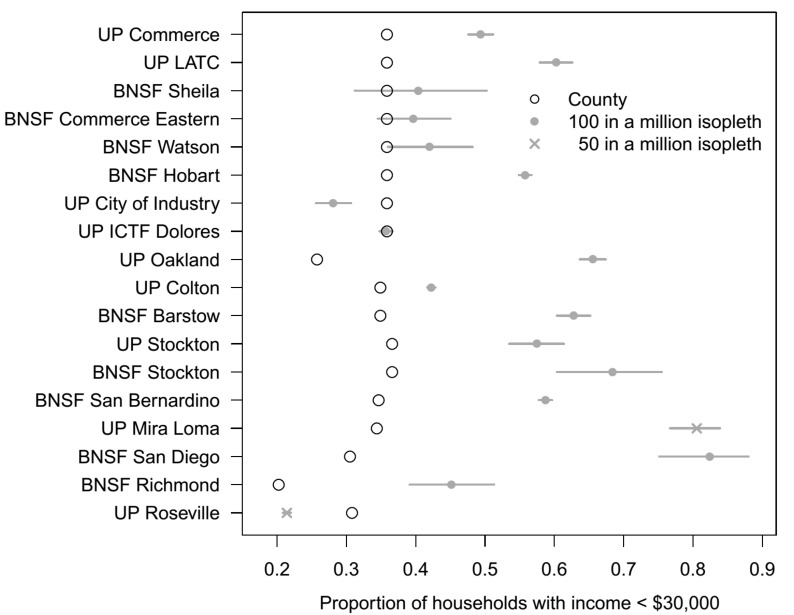
Estimated proportion ^1^ of low income households (<$30,000/year) living in close proximity to rail yards, where proximity is defined by a 50 in a million risk isopleth or 100 in a million risk isopleth, compared to the corresponding population proportions for the county in which the rail yard is located, with the rail yards listed in decreasing order of population proportion non-white in the County.

^1^ Point estimates are plotted along with 95% confidence intervals, though for most isopleths the sample size is large enough that the confidence intervals are very narrow and not visible at this scale.

With regard to income, as shown in Figure 5, the estimated percentage of low income households in the 100 in a million risk isopleth was higher than the population percentage of the county for 14 of the 16 rail yards (*p* *<* *0.0001 for the 11 rail yards where the 95% confidence intervals did not overlap with the population proportion, p* *=* *0.04 for BNSF Watson, p* *=* *0.15 for BNSF Commerce Eastern, and p* *=* *0.37 for BNSF Sheila*). For example, near UP Mira Loma (based on 50 in a million risk isopleth), the percentage of low income households in close proximity to the rail yard was 81% *vs*. 34% in the county; near UP Oakland the percentage of low income households in close proximity to the rail yard was 66% *vs.* 26% in the county. Only two rail yards (UP City of Industry and UP Roseville) had a smaller proportion of low income households in close proximity to the rail yards than in the County as a whole (*statistically significantly lower, p* *<* *0.0001*).

### 5.1. Which Came First, Siting of the Rail Yards or the Lower-Income Minority Populations Living in the Area? A Brief Case Study

**Figure 6:**
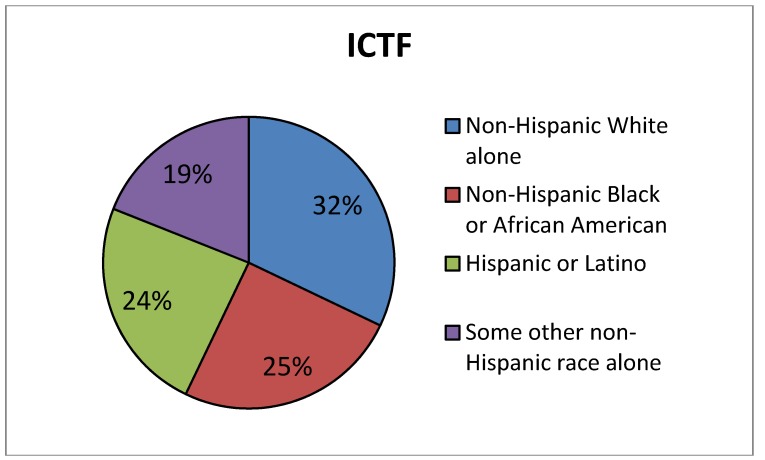
Demographics of the population in west Long Beach in close proximity to the ICTF rail yard, 1980 Census data^ a,b^.

^a^ Race/ethnicity percentages listed in decreasing order. Demographics by race/ethnicity listed in order of highest percentage. ^b^ We used the 100 in a million diesel cancer risk isopleths for this calculation. The footprint of the ICTF has not changed since 1980, so to be consistent with our other analyses we considered “in close proximity” to the ICTF to be residents living within the current ICTF 100 in a million isopleth.

**Figure 7:**
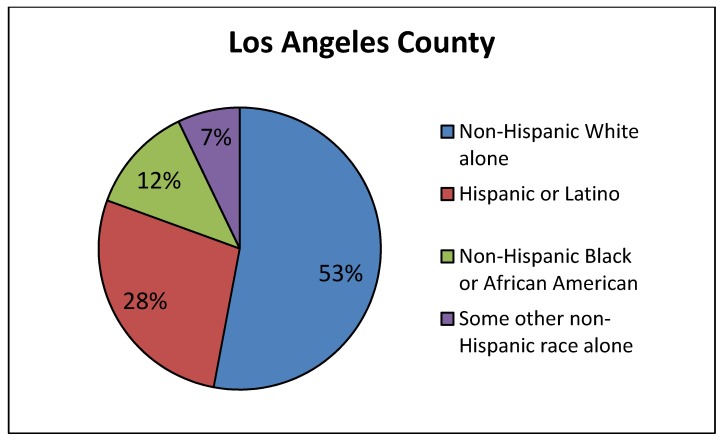
Demographics of the population in Los Angeles County, 1980 Census data.

*Site selected for the Union Pacific ICTF in the 1980s.* Many of the California rail yards were sited decades ago, making it difficult to determine whether the existing rail yards were built first or if the community might have settled there before the yard was built. One California rail yard, however, the UP ICTF, was approved and constructed within the past 30 years [49], so we conducted a demographic analysis of the population near that yard based on the 1980 census. Our results show that the nearby residents at the time the facility was debated and then approved were predominantly lower-income and minority, Figure 6. The figure shows that the estimated population percentage near the proposed UP ICTF based on 1980 Census data was only 32% White, while the White population percentage in Los Angeles County at that time was 53%, Figure 7. To look at it another way, at the time the rail yard project was approved, 68% of the nearby population was minority compared to 47% in the County as a whole. In addition, Figure 6 shows that the population percentage for African-Americans in the 1980 census was 25% near the proposed UP ICTF, higher than the percentage of African-Americans (12%) in Los Angeles County, Figure 7.

In Figure 6 and Figure 7 there are large differences between the area near the proposed UP ICTF and the County in the demographic category called “some other non-Hispanic race alone” from the 1980 census. Because the census at the time did not differentiate among Asians and Pacific Islanders, the large number of Filipino, Thai, Samoan, Tongan and other Asian Pacific Islanders living in west Long Beach in 1980 were combined into this generic category. 

The analyses showed that median income in the area of west Long Beach near the proposed UP ICTF, according to the 1980 Census, was $8,616 while the median income for Los Angeles County residents as a whole was more than twice this amount, $19,486. Thus, when the ICTF was built in 1986, the nearby community was also significantly lower-income than Los Angeles County as a whole.

In summary, at the time that the Ports of Los Angeles and Long Beach—and the railroad (at the time, Southern Pacific, today Union Pacific)—made the site selection for the ICTF, the demographic data were clear that the rail yard facility would be constructed adjacent to a working class/working poor community of color. The community was established in the location before the UP ICTF was built.

Issues of race and income of nearby residents were not mentioned in comments and letters submitted when the environmental impact reports were being prepared, but some residents of a nearby mobile home park (which still exists) raised concerns about future air pollution from the rail yard. The Final Environmental Impact Report (EIR) in 1986 concluded that: “Air quality impacts of the ICTF on adjacent residential areas are anticipated to be insignificant [48]”. Just 22 years later, CARB estimated that the UP ICTF was one of the four most polluting rail yards in the State of California, creating an estimated diesel cancer risk of greater than 100 in a million for more than 33,540 nearby residents, Table 2. 

**Figure 8:**
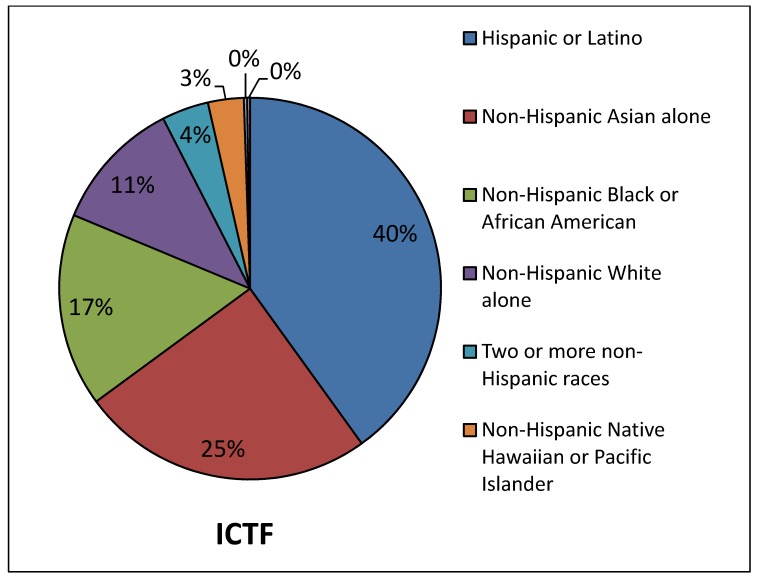
Demographics of the population in west Long Beach in close proximity to the ICTF rail yard, 2000 Census data and in Los Angeles County as a whole at the time that the railroad announced that it wanted to double its capacity; 2000 census data.

**Figure 9:**
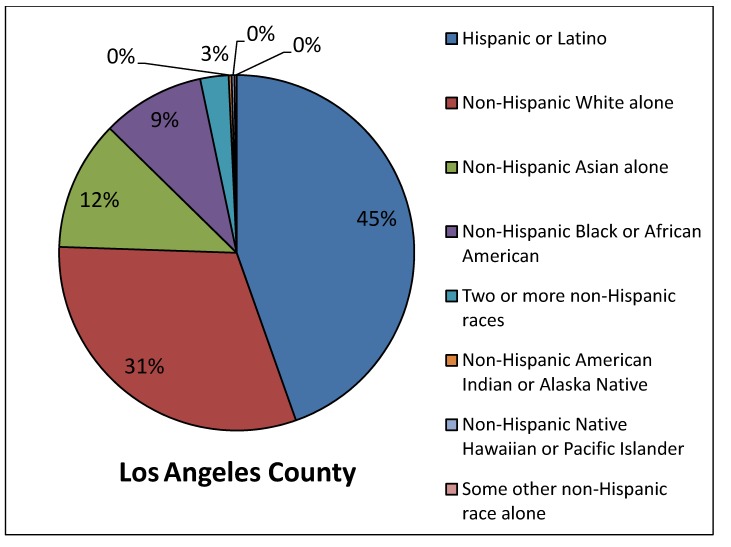
Demographics of the population in Los Angeles County at the time that Union Pacific announced it wanted to expand the UP ICTF rail yard, 2000 census data.

*Proposed UP ICTF expansion, 2005*. In 2005 Union Pacific announced that it wanted to expand the ICTF [49]. By that time, the population demographics had changed somewhat both near the ICTF and in Los Angeles County, but remained predominantly people of color, when compared to the County of Los Angeles as a whole (Figure 8 and Figure 9). In the 2000 census, only 11% of the residents living near the rail yard were White compared to 31% of Los Angeles County residents. In other words, the population around the ICTF when Union Pacific proposed to expand its rail yard in 2005, was 89% minority, compared to 69% of the County as a whole. The Union Pacific ICTF also has a significantly higher percentage of Asian Pacific Islanders near the yard, compared to the County as a whole (compare Figure 8 and Figure 9). For the area near the proposed UP ICTF, Asians/Asian Pacific Islanders comprised 28% of the nearby population in 2000, compared to 12% for the County.

A decision has not yet been made on expansion of the UP ICTF expansion project. The possible expansion is still being discussed by a Joint Powers Authority of the Ports of Los Angeles and Long Beach and is in the early environmental review process [49].

### 5.2. Construction of New Intermodal Facilities in the U.S. and Health/Environmental Concerns Raised by Residents

By reviewing the news media and industry trade journals, we identified multiple new intermodal rail facilities proposed to be built or recently constructed in the U.S. We looked at examples from the four largest Class I freight railroads in the country: Union Pacific (UP) (case study above); BNSF; CSX; and Norfolk Southern (NS). We discovered that siting rail yards close to homes and schools (a public health concern) or in lower-income minority communities (a public health and EJ concern) is not an historic artifact that ended decades ago; it is continuing today at some, although not all, new or proposed rail yards. Some examples where residents have raised questions about siting decisions, in addition to the UP ICTF already described, include:

- A proposed BNSF intermodal facility in Wilmington, CA (part of the City of Los Angeles) that would be located within 1,000 feet of schools, a daycare center and a housing complex and that would bring in thousands of trucks a day to the yard, which is four miles from the local ports; emissions and truck traffic would again impact the lower-income minority community of west Long Beach. The project, called the Southern California International Gateway (BNSF SCIG) was proposed in 2005 and had several iterations of an environmental impact report (EIR) between then and its final EIR in 2013 [50]. The location of this proposed rail yard is immediately south of the UP ICTF. Community residents and others raised public health and environmental justice concerns about building another rail yard in the same vicinity as the ICTF and in close proximity to homes and schools [51,52], urging that the rail yard be sited on-dock at the industrial ports rather than adjacent to a residential community. The Long Beach Unified School District [53] and others, including public health experts, also raised concerns about both of the proposed rail yards and their proximity to schools. Although BNSF Railway argues that the new rail yard would reduce regional pollution [54], an environmental report issued by the Port of Los Angeles on the project, under the California Environmental Quality Act (CEQA) stated that the impacts of localized air pollution from the rail yard:
  “*... would fall disproportionately on minority and low-income populations because the census block groups adjacent to the point of impact (the eastern edge of the Project site) constitute minority populations, and ... all or parts of [the adjacent] census tracts ... constitute low-income populations*.”
  [55]

**Figure 10:**
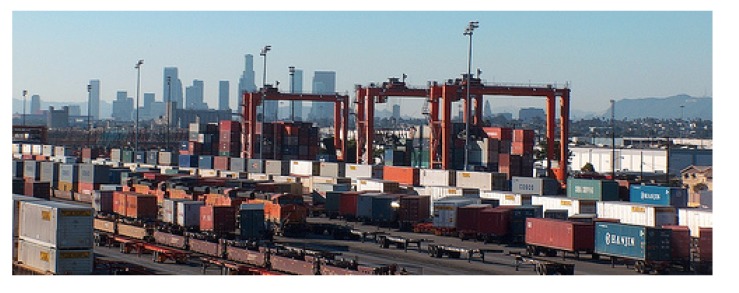
BNSF Hobart Yard, Commerce, CA with downtown Los Angeles in the background. Photo courtesy of Angelo Logan.

In 2013 the BNSF SCIG was approved by the Port of Los Angeles Harbor Commissioners and the City of Los Angeles [56]; there are multiple lawsuits against the project [57,58]. The new rail yard, if constructed, would be the second BNSF intermodal facility within 20 miles of the Ports of L.A. and Long Beach. BNSF’s Hobart Yard, Figure 10, located in Commerce, is the largest intermodal rail facility in the U.S.

**Figure 11:**
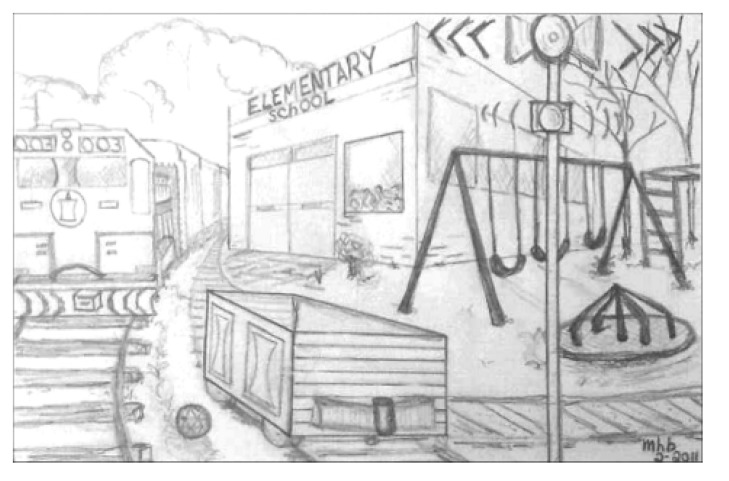
Sketch by Marihelen Ballard, Strawberry Plains, Tennessee, of a school in close proximity to a freight train traveling to a rail yard; reprinted with permission of the Jefferson County Post in New Market TN.

Selected other rail yard proposals or recently completed projects are described below:

- A Norfolk Southern (NS) rail yard newly constructed in Alabama that is immediately adjacent to an elementary school [59];
- A NS rail yard that is expanding by buying homes near its yard in a Chicago community called Englewood, home to mostly African-Americans [60];
- A CSX rail yard proposed in Baltimore, Maryland, that is estimated to bring 30–40 future trucks a day through a residential community [61]; and
- A NS rail yard proposed in a small town in Tennessee in close proximity to an elementary school, which prompted the following sketch, Figure 11, in a local newspaper as an indication of residents’ concerns [62].

**Figure 12:**
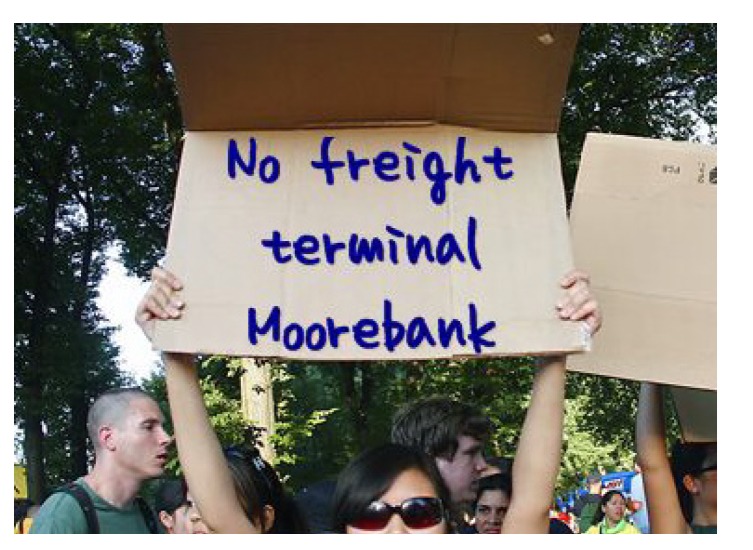
Australian residents in Liverpool protest proposal to build a freight terminal in nearby Moorebank, New South Wales (NSW).

The health concerns of residents near intermodal rail facilities are not limited to the U.S. In New South Wales, Australia, residents have raised concerns and protested plans to build a large intermodal rail facility at Moorebank to serve Port Botany (Figure 12). Port Botany is the second largest port in Australia, located 12 miles south of Sydney, with Moorebank being 22 miles southwest of Sydney. Residents in nearby Liverpool say they are concerned about truck traffic congestion, diesel emissions and noise that may come with the new intermodal rail yard [63].

Photo courtesy of Jim McGoldrick, Liverpool, Australia.

## 6. Discussion and Conclusions

Analyses by the California Air Resources Board, as estimated in the Rail Yard Health Risk Assessments, demonstrate that living in close proximity to rail yards with high levels of diesel exhaust emissions conveys a higher risk of cancer from diesel exhaust exposure than living a greater distance away from the source of pollution. The analyses allowed CARB to estimate differences in health risks among the rail yards, by drawing isopleths at various distances away from the yards to indicate differing risk levels. Besides distance, the isopleths took into account the direction and speed of the wind. Through that work, CARB was able to identify a number of yards in California that present particularly high diesel cancer risks for nearby residents. CARB also identified the City of Commerce as having seriously impacted residents, with four rail yards in one small community. We conclude that the siting of rail yards near sensitive receptors is a significant public health concern.

Our research utilized the CARB isopleths, 2000 Census data and GIS techniques to demonstrate significant diesel exposure disparities by race and income among residents living in close proximity to most of the 18 major freight rail yards in California where CARB has estimated high diesel cancer risks. We conclude that the location of existing or newly proposed rail yards in lower-income (working class/working poor) communities of color is a significant public health and environmental justice concern.

The analysis commissioned by EPA to support its locomotive engine rule investigated the populations living in close proximity to a representative sample of 37 U.S. rail yards, including three yards in California [64]. The EPA study found a large number of rail yards around the country with disproportionate impacts from diesel particulate matter at rail yards. For example, the EPA analysis states that “in Chicago the population living adjacent to the Barr Rail Yard, which has the greatest exposure to diesel emissions from that yard, is 97 percent African American, while the general metropolitan area of Chicago is only 18 percent African American [65]. 

Rail yards were also a topic of discussion by the Goods Movement Work Group (Work Group) for U.S. EPA’s National Environmental Justice Advisory Committee (NEJAC), which issued a report in 2009 [66]. The Work Group report concluded that “environmental pollution from the movement of freight is becoming a major public health concern at the national, regional and community levels,” and its report cited a U.S. EPA Inspector General’s report on the need to reduce air pollution for populations living near large diesel emission sources such as major roadways, rail yards, and ports, which are likely to experience greater diesel exhaust exposure levels than the overall U.S. population, exposing them to greater health risk [67].

In addition, Health Impact Assessments (HIAs) are a relatively new public health tool to assess impacts of proposed projects or policies [68]. The first HIA of an intermodal rail facility was recently published by the National Center for Healthy Housing; it examined the potential impacts of an expanded CSX rail facility to be constructed near the Port of Baltimore [69].

## 7. Recommendations

To protect residents, school children and EJ communities from environmental health impacts related to rail yards, we offer six policy recommendations for consideration:

1. Research. Conduct more epidemiologic research on the health and community impacts of rail yard facilities on nearby communities, additional exposure assessment studies, and evaluation of zero emission technologies for locomotives, trucks and rail yard equipment.
2. Best practices. Encourage the U.S. EPA to develop a best practices database for how to reduce air pollution at rail yards, including the availability of alternative technologies such as electric trucks and electric cranes, as recommended in the NEJAC Working Group report [66].
3. Siting and land use.
  - Whenever feasible, site rail yards servicing marine ports “on-dock” (that is, right at the marine terminals) in order to make the yards as efficient as possible and minimize the use of diesel-fueled drayage trucks.
  - Require minimum distances between rail yards and schools/homes and other sensitive receptors when choosing sites for new or expanded rail yards, taking into account CARB and other land use guidelines [19].
4. Environmental justice considerations.
  - Require that newly proposed rail yard facilities comply with Environmental Justice (EJ) Executive Orders and the EJ requirements of the U.S. Department of Transportation, U.S. Environmental Protection Agency and any state EJ directives, as relevant [70].
  - Discontinue to site rail yards in lower income, minority communities in favor of more suitable locations, including on-dock rail and purely industrial locations, in order to protect public health and uphold environmental justice principles.
5. Environmental reviews.
  - Require full Environmental Impact Statements under federal law or full reviews under state law, rather than simple Environmental Assessments when evaluating the impacts of major intermodal rail facilities.
  - Consider conducting Health Impact Assessments of any new rail yard facilities that are within one mile of homes and schools.
  - Require that all environmental reviews include a comparative demographic analysis (including race/ethnicity/income/educational attainment levels) of the neighborhoods within one mile of a proposed rail yard and the city/county as a whole and that the results of this analysis be included in the environmental statement or report.
  - Require that any environmental reviews of rail yard proposals include accurate forecasts for future truck and locomotive volumes; accurate assessments of projected emissions from trucks, locomotives and yard equipment; accurate assumptions in modeling of the near-roadway air pollution exposures; and an evaluation of alternative technologies; and that new projects adhere to what was promised in the environmental review reports.
6. Regulatory agencies.
  - Require that regulatory agencies with responsibility for air pollution from rail yard facilities (including locomotives and other equipment) have *mandatory* mechanisms in place to reduce public health risks when analyses or HRAs show elevated cancer or other health risks from exposure to diesel exhaust or other pollutants.
  - Update EPA’s assessment of diesel exhaust exposure’s health effects to reflect IARC’s designation of diesel exhaust as a “human carcinogen”.

Other promising policies and solutions that can be considered to reduce air pollution emissions from rail yards are described in a report by THE Impact Project, including (1) strengthening federal regulation of locomotives, with a goal toward zero-emission technologies; (2) seeking federal authority to allow additional state and local authority to address air pollution from rail yards; (3) allowing rail yards to be regulated as stationary sources so that local air regulators have the ability to demand emission reductions and idling control; and (4) requiring that the equipment used at rail yards use the maximum achievable air pollution control technology to reduce diesel emissions [71].

## Abbreviations

BNSF: BNSF Railway Company, formerly Burlington Northern Santa Fe Railway
CA: California
CARB: California Air Resources Board
CSX: CSX Corporation
DPM: diesel particulate matter
EJ: environmental justice
HRA: Health Risk Assessment
IARC: International Agency for Research on Cancer
ICTF: intermodal container transfer facility
UP: Union Pacific Railroad
U.S.: United States

## Supplementary Files

Supplementary File 1 Supplementary Information (PDF, 121 KB)
