# Supplementary material for: Global Trade, Local Impacts: Lessons from California on Health Impacts and Environmental Justice Concerns for Residents Living near Freight Rail Yards
Source: Int J Environ Res Public Health. 2014 Feb 10;11(2):1914–41. doi: 10.3390/ijerph110201914 (PMC3945577; doi:10.3390/ijerph110201914)
Supplement: Supplementary File 1 — Supplementary Information (PDF, 121 KB) [file ijerph-11-01914-s001.pdf]

Supplementary Information

Global Trade, Local Impacts: Lessons from California on Health Impacts and Environmental Justice Concerns for Residents Living near Freight Rail Yards

Table S1. List of rail yards with county and population proportion non-white in the corresponding county.

Rail yard	County	Proportion non-white in County
UP Commerce	Los Angeles	0.691
UP LATC	Los Angeles	0.691
BNSF Sheila	Los Angeles	0.691
BNSF Commerce Eastern	Los Angeles	0.691
BNSF Watson	Los Angeles	0.691
BNSF Hobart	Los Angeles	0.691
UP City Of Industry	Los Angeles	0.691
UP ICTF Dolores	Los Angeles	0.691
UP Oakland	Alameda	0.592
UP Colton	San Bernardino	0.562
BNSF Barstow	San Bernardino	0.562
UP Stockton	San Joaquin	0.528
BNSF Stockton	San Joaquin	0.528
BNSF San Bernardino	San Bernardino/Riverside [1]	0.528
UP Mira Loma	Riverside	0.491
BNSF San Diego	San Diego	0.451
BNSF Richmond	Contra Costa	0.423
UP Roseville	Placer	0.380

[1] The 100 in a million risk isopleth for BNSF San Bernardino is located in both San Bernardino and Riverside counties.

Table S2. Estimated proportion of African American or Hispanic/Latino residents living in close proximity to rail yards (where proximity is defined by 100 in a million risk isopleth), compared to the corresponding population proportions in the county in which the rail yard is located. The rail yards are listed in decreasing order of population proportion non-white in the County.

Location	Proportion African American			Proportion Hispanic/Latino		
	County	100 in a million isopleth		County	100 in a million isopleth	
		Estimate	95% CI		Estimate	95% CI
UP Commerce	0.094	0.003	0.002, 0.004	0.446	0.966	0.963, 0.969
UP LATC	0.094	0.209	0.202, 0.216	0.446	0.577	0.568, 0.585
BNSF Sheila	0.094	0.008	0.002, 0.025	0.446	0.894	0.857, 0.922
BNSF Commerce Eastern	0.094	0.011	0.006, 0.019	0.446	0.891	0.871, 0.908
BNSF Watson	0.094	0.047	0.036, 0.062	0.446	0.943	0.928, 0.956
BNSF Hobart	0.094	0.003	0.003, 0.004	0.446	0.973	0.972, 0.975
UP City of Industry	0.094	0.018	0.015, 0.022	0.446	0.829	0.820, 0.839
UP ICTF Dolores	0.094	0.164	0.160, 0.168	0.446	0.401	0.395, 0.406
UP Oakland	0.144	0.639	0.628, 0.649	0.190	0.189	0.180, 0.198
UP Colton	0.086	0.085	0.083, 0.087	0.392	0.666	0.663, 0.669
BNSF Barstow	0.086	0.089	0.080, 0.098	0.392	0.487	0.472, 0.503
UP Stockton	0.063	0.182	0.167, 0.199	0.305	0.638	0.617, 0.658
BNSF Stockton	0.063	0.108	0.085, 0.136	0.305	0.585	0.544, 0.625
BNSF San Bernardino	0.074	0.118	0.114, 0.121	0.378	0.738	0.734, 0.743
UP Mira Loma *	0.060	0.075	0.053, 0.103	0.362	0.416	0.372, 0.462
BNSF San Diego	0.054	0.175	0.147, 0.206	0.267	0.346	0.310, 0.384
BNSF Richmond	0.090	0.206	0.178, 0.237	0.177	0.535	0.498, 0.572
UP Roseville *	0.080	0.020	0.019, 0.021	0.149	0.123	0.121, 0.126

* UP Mira Loma and UP Roseville estimates and 95% CI are from 50 in a million isopleths.

Table S3. Estimated proportion of low income households (<$30,000/year) living in close proximity to rail yards (where proximity is defined by 100 in a million risk isopleth), compared to the corresponding population proportions for the county in which the rail yard is located, with the rail yards listed in decreasing order of population proportion non-white in the County.

Location	County	100 in a million isopleth	
		Estimate	95% CI
UP Commerce	0.358	0.494	0.476, 0.512
UP LATC	0.358	0.603	0.579, 0.626
BNSF Sheila	0.358	0.404	0.312, 0.502
BNSF Commerce Eastern	0.358	0.397	0.345, 0.450
BNSF Watson	0.358	0.420	0.360, 0.482
BNSF Hobart	0.358	0.558	0.549, 0.567
UP City of Industry	0.358	0.281	0.256, 0.307
UP ICTF Dolores	0.358	0.357	0.347, 0.367
UP Oakland	0.258	0.656	0.637, 0.674
UP Colton	0.349	0.422	0.416, 0.429
BNSF Barstow	0.349	0.628	0.604, 0.652
UP Stockton	0.366	0.575	0.535, 0.614
BNSF Stockton	0.366	0.684	0.604, 0.755
BNSF San Bernadino	0.346	0.587	0.578, 0.597
UP Mira Loma *	0.344	0.805	0.767, 0.839
BNSF San Diego	0.305	0.824	0.751, 0.880
BNSF Richmond	0.202	0.451	0.391, 0.513
UP Roseville *	0.308	0.214	0.208, 0.220

* UP Mira Loma and UP Roseville estimates and 95% CI are from 50 in a million isopleths.

© 2014 by the authors; licensee MDPI, Basel, Switzerland. This article is an open access article distributed under the terms and conditions of the Creative Commons Attribution license (http://creativecommons.org/licenses/by/3.0/).
